# A novel genomic panel as an adjunctive diagnostic tool for the characterization and profiling of breast Fibroepithelial lesions

**DOI:** 10.1186/s12920-019-0588-2

**Published:** 2019-10-23

**Authors:** Yirong Sim, Gwendolene Xin Pei Ng, Cedric Chuan Young Ng, Vikneswari Rajasegaran, Suet Far Wong, Wei Liu, Peiyong Guan, Sanjanaa Nagarajan, Wai Yee Ng, Aye Aye Thike, Jeffrey Chun Tatt Lim, Nur Diyana Binte Md Nasir, Veronique Kiak Mien Tan, Preetha Madhukumar, Wei Sean Yong, Chow Yin Wong, Benita Kiat Tee Tan, Kong Wee Ong, Bin Tean Teh, Puay Hoon Tan

**Affiliations:** 1 Division of Surgical Oncology, National Cancer Centre Singapore, Singapore, Singapore; 2 SingHealth Duke-NUS Breast Centre, Singapore General Hospital, Singapore, Singapore; 3 Integrated Genomics Platform, National Cancer Centre Singapore, Singapore, Singapore; 4 Laboratory of Cancer Epigenome, National Cancer Centre Singapore, Singapore, Singapore; 5 Integrated Biostatistics and Bioinformatics Program, Duke-NUS Medical School, Singapore, Singapore; 6 Duke-NUS Medical School, Singapore, Singapore; 7 Department of Anatomical Pathology, Singapore General Hospital, Singapore, Singapore; 8 Division of Pathology, Singapore General Hospital, Singapore, Singapore

**Keywords:** Breast, Fibroepithelial lesion, Fibroadenoma, Phyllodes, Genomic test, Core biopsy

## Abstract

**Background:**

Known collectively as breast fibroepithelial lesions (FELs), the common fibroadenomas (FAs) and the rarer phyllodes tumors (PTs) are a heterogenous group of biphasic neoplasms. Owing to limited tissue availability, inter-observer variability, overlapping histological features and heterogeneity of these lesions, diagnosing them accurately on core biopsies is challenging. As the choice management option depends on the histological diagnosis; a novel 16-gene panel assay was developed to improve the accuracy of preoperative diagnosis on core biopsy specimens.

**Methods:**

Using this 16-gene panel, targeted amplicon-based sequencing was performed on 275 formalin-fixed, paraffin-embedded (FFPE) breast FEL specimens, archived at the Singapore General Hospital, from 2008 to 2012.

**Results:**

In total, 167 FAs, 24 benign, 14 borderline and 6 malignant PTs, were profiled. Compared to FAs, PTs had significantly higher mutation rates in the *TERT* promoter (*p* <  0.001), *RARA* (*p* <  0.001), *FLNA*, *RB1* and *TP53* (*p* = 0.002, 0.020 and 0.018, respectively). In addition to a higher mutational count (*p* <  0.001), *TERT* promoter (*p* <  0.001), frameshift, nonsense and splice site (*p* = 0.001, < 0.001 and 0.043, respectively) mutations were also frequently observed in PTs.

A multivariate logistic regression model was built using these as variables and a predictive scoring system was developed. It classifies a FEL at low or high risk (score <  1 and ≥ 1, respectively) of being a PT. This scoring system has good discrimination (ROC area = 0.773, 95% CI: 0.70 to 0.85), calibration (*p* = 0.945) and is significant in predicting PTs (*p* <  0.001).

**Conclusion:**

This novel study demonstrates the ability to extract DNA of sufficient quality and quantity for targeted sequencing from FFPE breast core biopsy specimens, along with their successful characterization and profiling using our customized 16-gene panel. Prospective work includes validating the utility of this promising 16-gene panel assay as an adjunctive diagnostic tool in clinical practice.

## Background

Breast fibroepithelial lesions (FELs) belong to a family of biphasic neoplasms, characterized by the proliferation of both epithelial and stromal components. The common benign fibroadenomas (FAs) are distinguished from the much rarer phyllodes tumors (PTs) by the presence of leaf-like stromal fronds and increased stromal cellularity in the latter. Phyllodes tumors, which comprise less than 1% of all breast tumors, can be classified into benign (BEN), borderline (BDR) and malignant (MAL) grades based on the assessment of five histological parameters—stromal cellularity and overgrowth, nuclear atypia, cellular pleomorphism, mitotic activity and tumor borders [1, 2]. Compared to western counterparts, a higher incidence rate of PTs is observed in women of Asian descent [2–4].

Core needle biopsies are recommended for preoperative histological diagnoses of breast lesions as this technique is minimally invasive, cost-effective and can be carried out in an outpatient setting [5, 6]. In the context of breast FELs, the choice therapeutic option hinges on the histological diagnosis—either observation or enucleation for benign FAs, to a surgical resection with wide margins (wide excision) for PTs [6–8]. However, discriminating between the two entities preoperatively, in particular cellular FELs, can be challenging, especially with limited available material from core biopsies, due to overlapping histological features and lesional heterogeneity, compounded by inter-observer variability [8–11]. Thus, there is a need for a highly specific and sensitive pre-operative histopathologic diagnosis based on the limited material from core biopsies to avoid over- or under-treatment and to reduce unwarranted anxiety and cost to the patient [12]. Several studies have explored the genomic landscapes of breast FELs. *MED12* is the only gene that has mutations occurring frequently in FAs and PTs of all grades [13, 14], suggesting a biological link between these FELs that share morphological features and genetic abnormalities [15]. Other mutations observed more commonly in PTs consist of *RARA*, *FLNA*, *SETD2*, *KMT2D* and the *TERT* promoter gene abnormalities [16]. The frequency of mutations in the *TERT* promoter was observed to increase with increasing grade of PTs, implying its possible role in driving the progression of PTs and differentiating FAs from the PTs [17, 18]. Other oncogenes associated with the borderline and malignant spectrum of PTs include *TP53*, *RB1*, *EGFR* and *NF1* [16–21].

Recently, Lucence Diagnostics released the FibroPhyllo™ Tissue Test [22], developed from a 5-gene reverse transcription-PCR assay (which measures the expression of *ABCA8, APOD, CCL19, FN1* and *PRAME*) [12], to augment the pathological distinction between FAs and PTs in breast lumps. Working on the common genes involved across the FEL spectrum, our group developed a 16-gene genomic assay to characterize FELs on core biopsy material using Next Generation Sequencing (NGS) [16]. The genes include the frequently observed MED12 and RARA mutations in both fibroadenomas and phyllodes, as well as the mutations in FLNA, SETD2, KMT2D,TERT promoter, NF1, RB1, TP53, PIK3CA, ERBB4 and EGFR which are seen more in the phyllodes tumors. This 16-gene genomic assay has been used anecdotally in selected cases to aid histopathologically challenging cases, such as the grading of phyllodes tumors [23], and where a malignant spindle cell tumors posed a diagnostic dilemma, mimicking metaplastic breast carcinoma [23, 24]. This novel 16-gene targeted panel [16, 23, 24], curated based on their likely involvement in fibroepithelial tumorigenesis, combined with a predictive model, has the potential to serve as an adjunctive diagnostic tool, refining indeterminate histological diagnoses in FELs, particularly on core biopsies.

## Methods

### Clinical specimens and diagnostic criteria

**Fig. 1:**
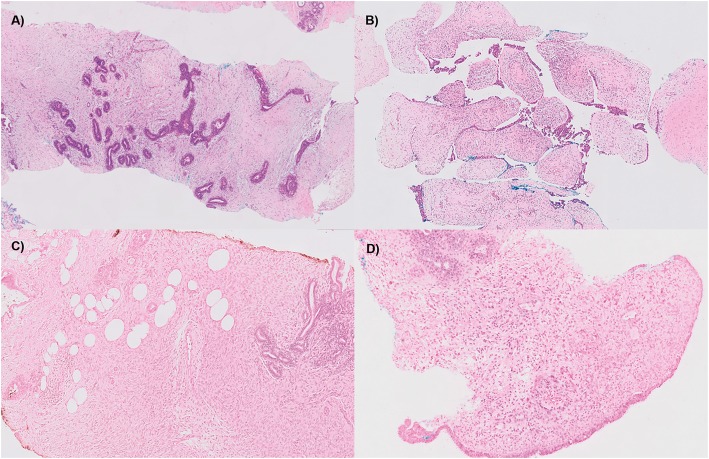
Histological features of representative core needle biopsies of **a**) fibroadenoma, **b**) benign, **c**) borderline and **d**) malignant phyllodes tumors confirmed on subsequent excisions

This study was conducted with the approval of the Centralized Institutional Review Board (CIRB Ref: 2016/2819). A total of 275 FEL specimens, comprising 241 core biopsies and 34 surgical excisions, was randomly selected from cases diagnosed at the Department of Anatomical Pathology, Singapore General Hospital from 2008 to 2012. Twenty-four FELs were from 12 paired specimens, each comprising a core biopsy and a surgical excision removed from the same patient. Haematoxylin and eosin (H&E)-stained slides of formalin-fixed and paraffin-embedded (FFPE) samples were retrieved, pathologically examined and graded in accordance to recommendations of the World Health Organization Classification of Tumors of the Breast [25]. Examples of the histological features of each subtype are as shown in Fig. 1.

### DNA extraction and quality control

Core biopsies and representative areas of resected tumors were identified and cut into two 10-μm-thick sections. Deparaffinization of sections was carried out with changes of limonene and graded ethanol. These deparaffinized sections were subsequently air dried and used for genomic DNA (gDNA) extraction with the QIAamp DNA FFPE Tissue Kit (Qiagen, Germany) as per protocol. PicoGreen fluorometric analysis and multiplex PCR assay were used to determine the quantity and quality of the extracted gDNA respectively.

### Library preparation for downstream next generation sequencing

Amplicon-based sequencing libraries were prepared in accordance to the manufacturer’s instructions within the QIAseq Targeted DNA Panel Kit (Qiagen, United States), using a minimum of 50 ng of gDNA. The gDNA was fragmented prior to the ligation with QIAseq adapters. Post ligation, amplification of the ligated products was carried out using the custom panel comprising of 16 genes—*MED12*, *TERT* promoter (Chr 5:1295100–1,295,300), *KMT2D*, *RARA*, *SETD2*, *FLNA*, *NF1*, *EGFR*, *RB1*, *TP53*, *PIK3CA*, *BCOR*, *PTEN*, *ERBB4*, *MAP3K1* and *IGF1R* [16, 23, 24]. The amplified prepared library was subsequently sequenced to a depth of greater than 300X of the target regions on the Illumina Hiseq 4000, to generate 150 bp paired-end reads.

### Bioinformatics analysis and the validation of variants

A quality assessment of the raw reads was done using FastQC (version 0.11.5). The trimmed reads were obtained after removing the first 30 bp of reads (adapters) and were aligned to the human genome, hg 19, using BWA-MEM (version 0.7.15-r1140, default setting). Point mutations and indels were called using FreeBayes (version v1.1.0–4-gb6041c6, settings: -m30 -q30 -F0.01) and annotated with wANNOVAR (http://wannovar.wglab.org/). Variants were further filtered to remove synonymous variants and variants in dbSNP. Variants that did not have a minimum coverage of 100X or have allelic frequencies lower than 5% were excluded. The remaining variants were visually curated using the Integrative Genomics Viewer (IGV) 2.3 genome browser to further exclude possible germline mutations and sequencing artifacts [26]. The data has been uploaded on EMBL-EBI, European Nucleotide Archive (https://www.ebi.ac.uk/ena); ascension number: PRJEB34134.

### Statistical analyses

Demographics were reported using T Test and Fisher’s Exact Test for numerical and categorical variables respectively. The frequencies of mutations and types of mutations observed in the 16 genes were compared between FAs and the PTs using the Fisher’s Exact Test. The number of mutations observed in each sample, defined hereafter as the mutation count, were tabulated and the samples were subsequently categorized into two groups—three or more mutations and less than three mutations. Similarly, these two groups were compared using the Fisher’s Exact Test.

### The development of a predictive scoring system

Univariate logistic regression models were generated to determine the variables to be used in the construction of a multivariate logistic regression model. Variables were only included when their *p* <  0.10. The multivariate logistic regression model was built using backward selection. Predictors which attained *p* <  0.05 were subsequently included the final model. The beta coefficients of these predictors in the final model were transformed into risk scores by dividing the coefficients by the lowest value. The performance of this multivariate model was assessed by the area under the Receiver Operating Characteristics (AUROC) curve and Hosmer Lemeshow Goodness of Fit Test. The risk groupings were further assessed in a logistic regression model using the true diagnoses as an outcome. Statistical significance was defined as when *p* <  0.05. An internal validation of our prognostic model was performed using the Bootstrapping technique. All analyses were performed in Stata version 12.0 [27].

Five other classification models were attempted to determine the best prediction model and to develop a predictive scoring system - gradient boosting, random forest, decision tree, support vector machine and KN neighbour classifier. 10-fold cross validations for each model were performed and the accuracy score for each model was calculated using python.

## Results

### Demographics of the patients and characteristics of breast Fibroepithelial lesions

A total of 275 diagnosed FELs—212 FAs, 35 benign, 21 borderline and 7 malignant PTs—were analyzed in this study. Of these, 12 were paired biopsies (i.e. core biopsy and surgical excision from the same patient)—5 FAs, 3 benign, 2 borderline and 2 malignant PTs.

The mean ages of the 207 patients diagnosed with FAs and 56 patients diagnosed with PTs were 45.2 (standard deviation, SD = 12.3) and 47.6 years (SD = 11.9) old respectively. The higher than expected mean age of diagnosis of FAs in this study is likely due to a clinical and selection bias, where older women with breast lumps are preferentially selected to undergo a biopsy or an excision, in contrast to observation and active surveillance in the younger population. There were no significant differences observed between these two groups of patients with respect to age (*p* = 0.189) and ethnicity (*p* = 0.101; Table 1).

**Table 1:** Demographics of patients with fibroepithelial lesions in this study

Features	Fibroadenoma (*n* = 207) ^**Ɨ**^	Phyllodes Tumor (*n =* 56) ^**Ɨ**^	*p* value
Age (mean, SD) ^a^	45.2, 12.3	47.6, 11.9	0.189
Ethnicity (*n*, %) ^b^			
Chinese	152 (73.4%)	33 (58.9%)	0.101
Malay	17 (8.2%)	10 (17.9%)	
Indian	10 (4.8%)	4 (7.1%)	
Others	028 (13.5%)	09 (16.1%)	

^**Ɨ**^The surgical excisions of the paired biopsies were *excluded*

^a^ T Test, comparing the age distribution between the patients diagnosed with fibroadenomas (FA) and phyllodes tumor (PT)

^b^ Fisher’s Exact Test, comparing the ethnicity distribution between the patients diagnosed with FA and PT.

### An assessment of the quality and quantity of the extracted DNA

**Fig. 2:**
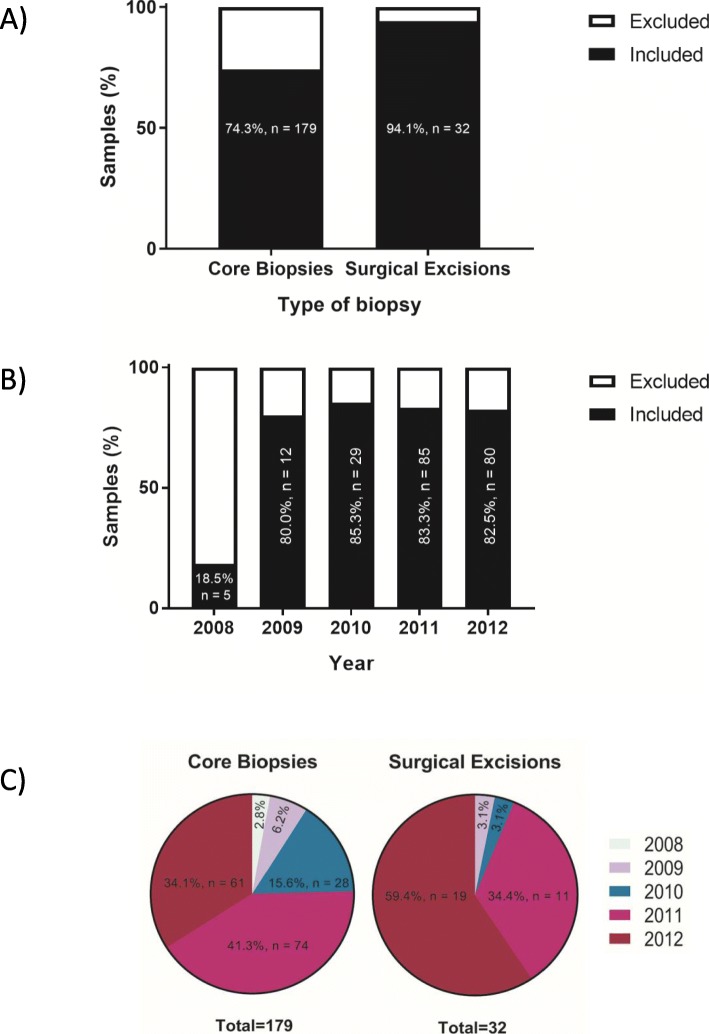
An assessment of the quality and quantity of the extracted DNA from 275 fibroepithelial lesions for downstream sequencing processes. The percentages of biopsy samples **a**) by types, **b**) by years which they were obtained and fixed, **c**) by both types and years that were suitable for use with the 16-gene genomic assay

DNA was extracted from the FFPE tissues of all 275 FELs, but only 74.3% (*n* = 179) and 94.1% (*n* = 32) of the 241 core biopsies and 34 surgical excisions, respectively, contained DNA of sufficient quality and quantity for targeted sequencing (Fig. 2a). As observed, the quality of the extracted gDNA tended to suffer as the number of archival years of the FFPE tissues increased. Excluding the 2008 samples, we achieved a high success rate of 81.3% in extracting good quality DNA of sufficient quantity from core biopsy specimens. Hence, good quality DNA that is suitable for downstream NGS, was effectively obtained from FFPE samples that were less than 7 to 8 years of age (Fig. 2b). Although there was a higher success rate of extracting good quality amplifiable DNA observed with surgical excision specimens, the majority of them were derived from recent years (Fig. 2c).

Of the 211 FELs that passed the quality and quantity assessment to proceed with downstream NGS were 167 FAs, 24 benign, 14 borderline and 6 malignant PTs. Amongst them were three paired biopsies that included a benign PT and two malignant PTs.

### Molecular profiling of the breast Fibroepithelial lesions

**Fig. 3:**
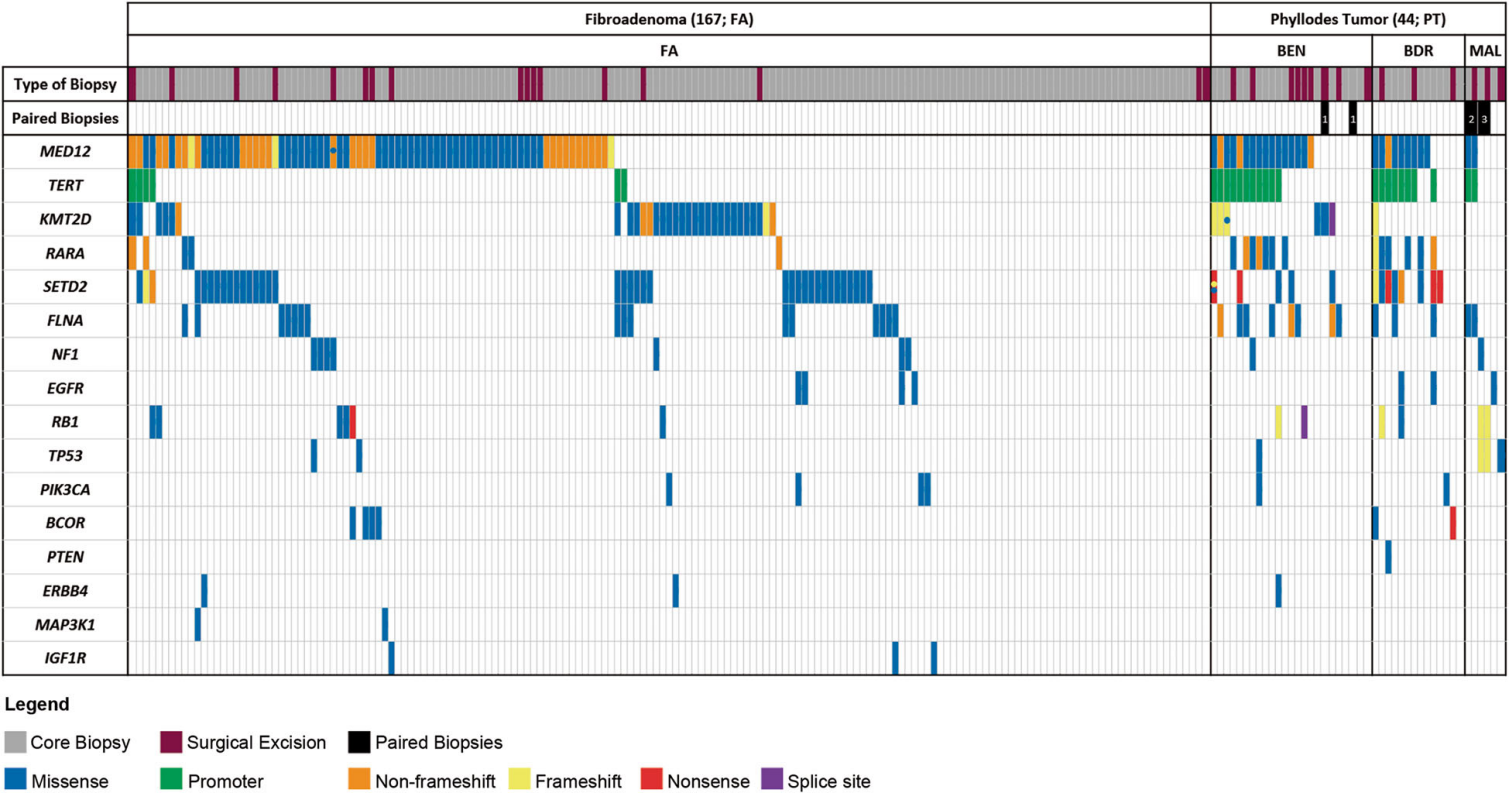
The genetic landscape of breast fibroepithelial lesions. The distribution of the recurrently mutated genes was identified through the targeted sequencing of 211 fibroepithelial lesions. Top, the method of biopsy is identified (core biopsy, grey; surgical excision, maroon). There were 179 core biopsies and 32 surgical excisions, of which, three were paired (paired biopsies, black). The histological diagnoses of the biopsies were as described: fibroadenoma (FA), phyllodes tumor (PT), benign (BEN) PT, borderline (BDR) PT and malignant (MAL) PT. In total, there were 167 FAs, 24 BEN PTs, 14 BDR PTs and 6 MAL PTs. The colored rectangles indicate the type of somatic mutations observed in the lesions. The colored dots signify additional mutations observed. The 16 genes used in the targeted sequencing of these lesions are listed on the left

A total of 321 mutations were seen in 164 samples, encompassing 125 FAs, 20 benign, 13 borderline and all 6 malignant PTs. No mutations were seen in the remaining 47 FELs, which included 42 FAs, 4 benign and 1 borderline PT (Fig. 3).

Mutations were observed in all 16 genes across the FELs, except for a lack of *PTEN* mutations in FAs and an absence of *MAP3K1* and *IGF1R* mutations in PTs. Common to all grades of PTs were mutations in *MED12*, *TERT* promoter, *FLNA* and *RB1*. In both benign and borderline PTs, mutations in *KMT2D*, *RARA*, *SETD2* and *PIK3CA* were present. Mutations in *NF1* and *TP53* were observed in both benign and malignant PTs. Common to both borderline and malignant PTs were mutations in *EGFR*. A mutation in *ERBB4* was seen in the benign PTs only; whereas, mutations in *BCOR* and *PTEN* were observed in the borderline PTs only (Fig. 3).

### Concordance of the molecular profiles of the paired biopsies

Although there were 12 paired biopsies retrieved for this study, only 3 pairs were suitable for analysis. Of these three pairs, only one pair of malignant PTs had an identical genetic profile in both the core biopsy and surgical excision specimens. They had mutations in *MED12*, *TERT* promoter and *FLNA* (Fig. 3). An analysis of the remaining two other pairs of biopsies showed the following: 1) the first was a pair of benign PTs, with the surgical excision possessing a mutation in *KMT2D* but with no mutations observed in the core biopsy; 2) the second pair of malignant PTs had mutations in *TP53* and *RB1* in both samples, but there was an additional mutation in the *NF1* gene observed in the core biopsy but not in the surgical excision (Fig. 3).

### TERT promoter, RARA, FLNA, RB1 and TP53 were more likely to be mutated in Phyllodes tumors

Most abnormalities occurred in codon 44 of exon 2 in *MED12*, the most frequently mutated gene amongst the FELs. *MED12* mutations were observed in 44.9 and 61.4% of FAs and PTs, respectively. The percentages of samples that exhibited *MED12* mutations were observed to decrease across the PT grades—66.7, 64.3 and 33.3% in benign, borderline and malignant PTs respectively (Table 2).

**Table 2:** Frequencies of mutations in the 16 genes, by FEL subtypes. Benign (BEN), borderline (BDR) and malignant (MAL) phyllodes tumor (PT)

Gene	Fibroadenoma (*n* = 167)		Phyllodes Tumor (*n* = 44)								*p* value
			BEN (*n* = 24)		BDR (*n* = 14)		MAL (*n* = 6)		All PT		
	*n*	*(%)*	*n*	*(%)*	*n*	*(%)*	*n*	*(%)*	*n*	*(%)*	
*MED12*	75	*(44.9)*	16	*(66.7)*	9	*(64.3)*	2	*(33.3)*	27	*(61.4)*	0.063
*TERT*	6	*(3.6)*	11	*(45.8)*	8	*(57.1)*	2	*(33.3)*	21	*(47.7)*	< 0.001
*KMT2D*	30	*(18.0)*	6	*(25.0)*	1	*(7.1)*	0	*(0.0)*	7	*(15.9)*	0.827
*RARA*	5	*(3.0)*	7	*(29.2)*	6	*(42.9)*	0	*(0.0)*	13	*(29.6)*	< 0.001
*SETD2*	36	*(21.6)*	5	*(20.8)*	8	*(57.1)*	0	*(0.0)*	13	*(29.6)*	0.316
*FLNA*	16	*(9.6)*	8	*(33.3)*	3	*(21.4)*	2	*(33.3)*	13	*(29.6)*	0.002
*NF1*	7	*(4.2)*	1	*(4.2)*	0	*(0.0)*	1	*(16.7)*	2	*(4.6)*	1.000
*EGFR*	4	*(2.4)*	0	*(0.0)*	2	*(14.3)*	1	*(16.7)*	3	*(6.8)*	0.160
*RB1*	6	*(3.6)*	2	*(8.3)*	2	*(14.3)*	2	*(33.3)*	6	*(13.6)*	0.020
*TP53*	2	*(1.2)*	1	*(4.2)*	0	*(0.0)*	3	*(50.0)*	4	*(9.1)*	0.018
*PIK3CA*	4	*(2.4)*	1	*(4.2)*	1	*(7.1)*	0	*(0.0)*	2	*(4.6)*	0.607
*BCOR*	4	*(2.4)*	0	*(0.0)*	2	*(14.3)*	0	*(0.0)*	2	*(4.6)*	0.607
*PTEN*	0	*(0.0)*	0	*(0.0)*	1	*(7.1)*	0	*(0.0)*	1	*(2.3)*	0.209
*ERBB4*	2	*(1.2)*	1	*(4.2)*	0	*(0.0)*	0	*(0.0)*	1	*(2.3)*	0.506
*MAP3K1*	2	*(1.2)*	0	*(0.0)*	0	*(0.0)*	0	*(0.0)*	0	*(0.0)*	1.000
*IGF1R*	3	*(1.8)*	0	*(0.0)*	0	*(0.0)*	0	*(0.0)*	0	*(0.0)*	1.000

The *TERT* promoter was the next most commonly mutated gene in PTs (Table 2). *TERT* promoter mutations were rare in FAs. In PTs, a mutation in the *TERT* promoter was often accompanied by a mutation in the *MED12* gene. Despite that, two FAs and a single PT displayed a mutation in the *TERT* promoter without a mutation in the *MED12* gene. Another set of 16 PTs—8 benign, 4 borderline and 4 malignant PTs lacked mutations in both *MED12* and the *TERT* promoter. However, these PTs had additional mutations in *KMT2D*, *SETD2*, *FLNA*, *PIK3CA*, *BCOR*, *NF1*, *EGFR*, *RB1* and *TP53* (Fig. 3).

There were significantly higher mutation rates in PTs than FAs in these genes—the *TERT* promoter (47.7% in PTs vs 3.6% in FAs; *p* <  0.001), *RARA* (29.6% in PTs vs 3.0% in FAs; *p* <  0.001), *FLNA* (29.6% in PTs vs 9.6% in FAs; *p* = 0.002), *RB1* (13.6% in PTs vs 3.6% in FAs; *p* = 0.020) and *TP53* (9.1% in PTs vs 1.2% in FAs; *p* = 0.018; Table 2).

### Phyllodes tumors have significantly higher mutation counts than Fibroadenomas

Of the total of 321 genetic alterations observed in our study, a total of 203 mutations were from FAs, and 118 mutations were from PTs (Fig. 3). PTs possessed significantly higher mutation counts than FAs, with a higher percentage of PTs having three or more mutations (54.6% in PTs vs 8.4% in FAs; *p* <  0.001) compared to FAs (Table 3). The FAs had a median of 1 mutation (mean = 1.2, range = 0 to 4), and the PTs had a median of 3 mutations (mean = 2.7, range = 0 to 7). However, there were no significant differences in the number of mutations observed between benign, borderline and malignant PTs (*p* = 1.000), implying that either the number of mutations gained may not have any implications on PT grade, or the sample size is too small which is likely the case.

**Table 3:** Fibroepithelial lesions were classified into two groups: 1) three or more mutations or 2) less than three mutations. The two groups were compared using the Fisher’s Exact Test

Mutation count	Fibroadenoma (*n* = 167)		Phyllodes Tumor (*n* = 44)		*p* value
	*n*	%	*n*	%	
< 3	153	91.6	20	45.5	< 0.001
≥ 3	14	8.4	24	54.6	

### Types of mutations observed in breast Fibroepithelial lesions

Applying our 16-gene assay on our samples, the following mutations were observed in FAs: missense, promoter, non-frameshift, frameshift and nonsense mutations. The following mutations were observed in PTs: missense, promoter, non-frameshift, frameshift, nonsense and splice site mutations (Fig. 3).

From the 167 FAs and 44 PTs sequenced, we discovered that missense mutations were the most common in both FAs and PTs (65.9% in PTs vs 77.3% in FAs). There were significantly more PTs having promoter mutations (47.7% in PTs vs 3.6% in FAs; *p* <  0.001), frameshift mutations (18.2% in PTs vs 3.0% in FAs; *p* = 0.001), nonsense mutations (13.6% in PTs vs 0.6% in FAs; *p* <  0.001) and splice site mutations (4.6% in PTs vs 0% in FAs; *p* = 0.043; Table 4).

**Table 4:** Types of mutations observed in 211 fibroepithelial lesions

Type of Mutation	Fibroadenoma (*n* = 167)		Phyllodes Tumor *(n* = 44)		*p* value
	*n*	*(%)*	*n*	*(%)*	
Missense	110	*(65.9)*	34	*(77.3)*	0.202
Promoter	6	*(3.6)*	21	*(47.7)*	< 0.001
Non Frameshift	33	*(19.8)*	10	*(22.7)*	0.676
Frameshift	5	*(3.0)*	8	*(18.2)*	0.001
Nonsense	1	*(0.6)*	6	*(13.6)*	< 0.001
Splice site	0	*(0.0)*	2	*(4.6)*	0.043

### A predictive scoring system as an adjunctive diagnostic tool for FELs

The univariate analyses identified ten predictors with *p* <  0.10: mutations in *MED12*, *TERT* promoter, *RARA*, *FLNA*, *RB1*, *TP53*; promoter, frameshift and nonsense mutations, as well as the possession of three or more mutations (Table 5). These ten variables were subsequently used to build a multivariate logistic regression model. The variables with *p* <  0.05 in the final model were mutations in *TP53* (OR = 13.54, 95% confidence interval, CI: 2.99 to 61.31), *TERT* promoter (OR = 24.10, 95% CI: 10.94 to 53.10) and nonsense mutation (OR = 19.75, 95% CI: 5.32 to 73.30; Table 6). The ROC of the model was 0.773, 95% CI: 0.70 to 0.85, which showed that this model had good discriminant ability. The Hosmer-Lemeshow Goodness of Fit Test indicated good calibration with *p* = 0.945. An internal validation of our prognostic model using the bootstrapping technique was carried out based on 796 replications providing the same coefficient estimates with ROC = 0.81, 95%CI (0.72 to 0.90). This demonstrates good discriminant ability between FA and PT, and the Hosmer Lemeshow goodness of fit test *p*-value was 0.973, indicating good calibration.

**Table 5:** A summary of the univariate analyses performed to understand the effect of each potential predictor in the classification of the fibroepithelial lesions. Predictors (^***Ɨ***^), with *p* < 0.10, were included in the multivariate analysis

Predictors	Odds Ratio (OR)	95% Confidence Interval (CI)	*p* value
*Genes*			
*MED12*^***Ɨ***^	1.95	0.99 to 3.84	0.054
*TERT promoter*^***Ɨ***^	24.50	8.95 to 67.07	< 0.001
*KMT2D*	1.10	0.46 to 2.60	0.836
*RARA*^***Ɨ***^	13.59	4.52 to 40.84	< 0.001
*SETD2*	1.53	0.72 to 3.22	0.266
*FLNA*^***Ɨ***^	3.96	1.73 to 9.06	0.001
*NF1*	1.09	0.22 to 5.43	0.918
*EGFR*	2.98	0.64 to 13.85	0.163
*RB1*^***Ɨ***^	4.24	1.29 to 13.86	0.017
*TP53*^***Ɨ***^	8.25	1.46 to 46.64	0.017
*PIK3CA*	1.94	0.34 to 10.96	0.453
*BCOR*	1.94	0.34 to 10.96	0.453
*PTEN*	Excluded, frequency < 5		
*ERBB4*	1.92	0.17 to 21.66	0.598
*MAP3K1*	Excluded, frequency < 5		
*IGF1R*	Excluded, frequency < 5		
*Mutation types*			
Missense	1.76	0.81 to 3.82	0.152
Promoter^**Ɨ**^	24.50	8.95 to 67.07	< 0.001
Non Frameshift	1.19	0.54 to 2.67	0.664
Frameshift^**Ɨ**^	7.20	2.23 to 23.30	0.001
Nonsense^**Ɨ**^	26.20	3.06 to 224.10	0.003
Splice site	Excluded, frequency < 5		
*Mutation count*			
Mutation count (≥3) ^**Ɨ**^	13.11	5.85 to 29.40	< 0.001

**Table 6:** A summary of the predictors whose *p* < 0.05 that were included in the final multivariate logistic regression model

Predictors	Odds Ratio (OR)	95% Confidence Interval (CI)	β coefficient	Standard Error (SE)	*p* value
*Genes*					
*TP53*	13.54	2.99 to 61.31	2.61	0.94	0.001
*Mutation types*					
Promoter	24.10	10.94 to 53.10	3.18	0.63	< 0.001
Nonsense	19.75	5.32 to 73.30	2.98	0.77	< 0.001

In addition to the logistic regression model, five other classification models were applied to determine the best prediction model for a predictive scoring system. These include gradient boosting, radnom forest, decision tree, support vector machine and KN neighbour classifier. A 10 fold cross validation was performed for all 6 models, and the accuracy scores were calculated and compared (Table 7). With the highest accuracy score, the logistic regression model was then adopted in our prediction model.

**Table 7:** A comparison of the accuracy scores across the 6 different classification models (statistical and machine learning techniques)

Model	Accuracy Score
Logistic Regression	0.87
Gradient Boosting	0.85
Random Forest	0.82
Decision Tree	0.76
Support Vector Machine	0.77

The beta coefficients of these chosen predictors in the final model were used to calculate the risk scores. Presence of *TP53* mutation, promoter and nonsense mutations each contributed 1, 1.22 and 1.14 points, respectively. The risk score was classified into two groups: i) low risk (< 1 point) and ii) high risk (≥ 1 point) of being a PT (Table 8). Logistic regression analysis showed that these groupings significantly predicted PTs with *p* <  0.001; those in the high risk group were 25 times more likely to have PT compared to the low risk group.

**Table 8:** The scorecard describing the weightage points of each predictor that was derived through their beta coefficients and the cut-off points required for a lesion to be classified as either a fibroadenoma or a phyllodes tumor

Predictors	Score
*Genes*	
*Presence of mutations in TP53 gene*	
Yes	1
No	0
*Mutation types*	
Presence of promoter mutation	
Yes	1.22
No	0
Presence of nonsense mutation	
Yes	1.14
No	0
*Risk groups*	
Low risk of being a phyllodes tumor	< 1
High risk of being a phyllodes tumor	≥ 1

**Fig. 4:**
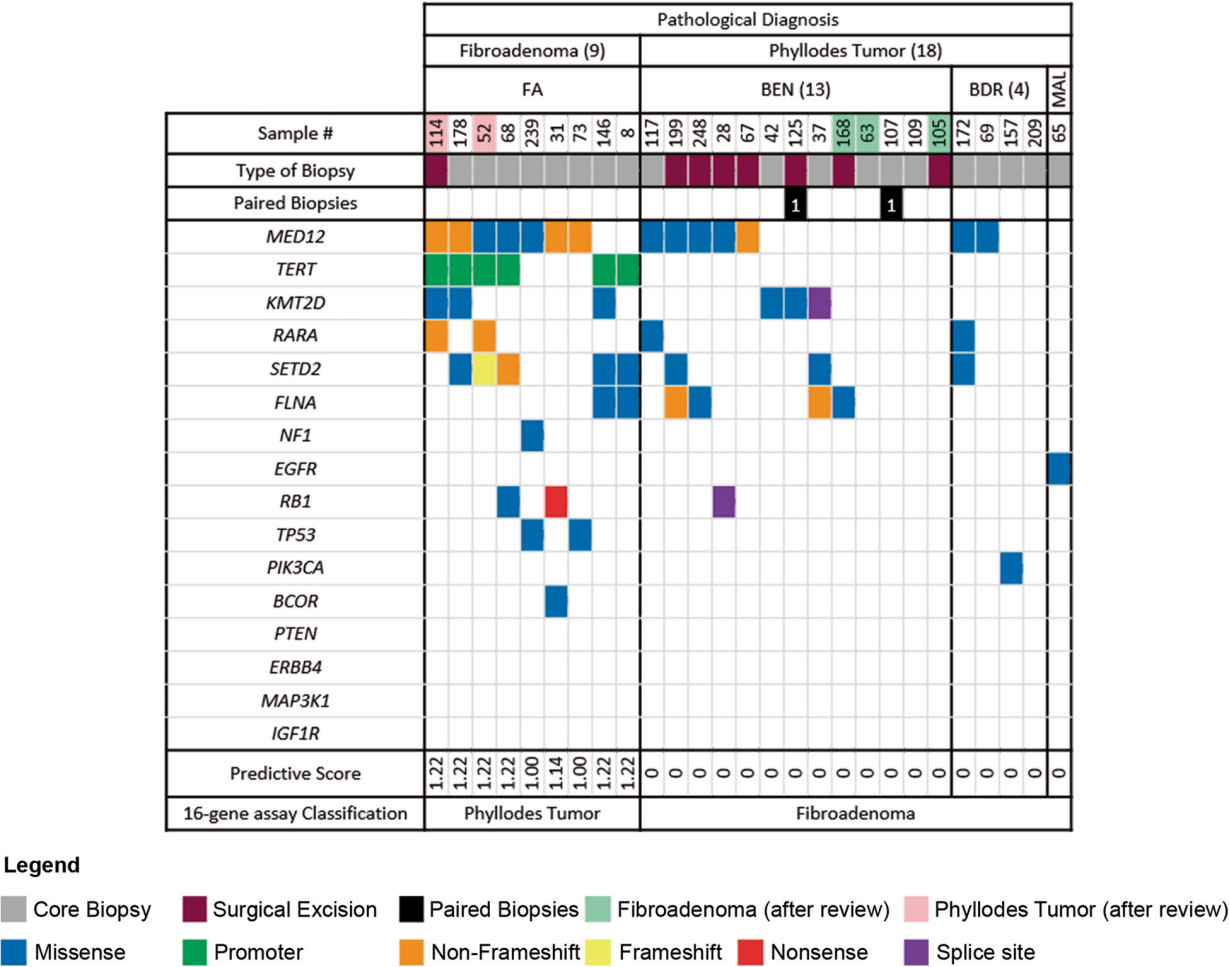
The genetic landscape of the 27 discordant cases, comprising 9 fibroadenomas (FAs) and 18 phyllodes tumors (PTs). This figure demonstrates the discrepancies between the original pathological diagnoses of the FELs and the diagnoses of the fibroepithelial lesions based on the predictive scoring model and the 16-gene assay. Top, pathological diagnoses, the sample identification number and type of biopsy material. Samples that have their diagnoses changed on second pathology review are highlighted. Left, the 16 genes used in the targeted sequencing of these lesions are listed. The colored rectangles indicate the type of somatic mutations observed in the lesions through the 16-gene assay. Bottom, the respective predictive scores calculated based on the scorecard (Table 6) and their corresponding classifications by the 16-gene assay

**Fig. 5:**
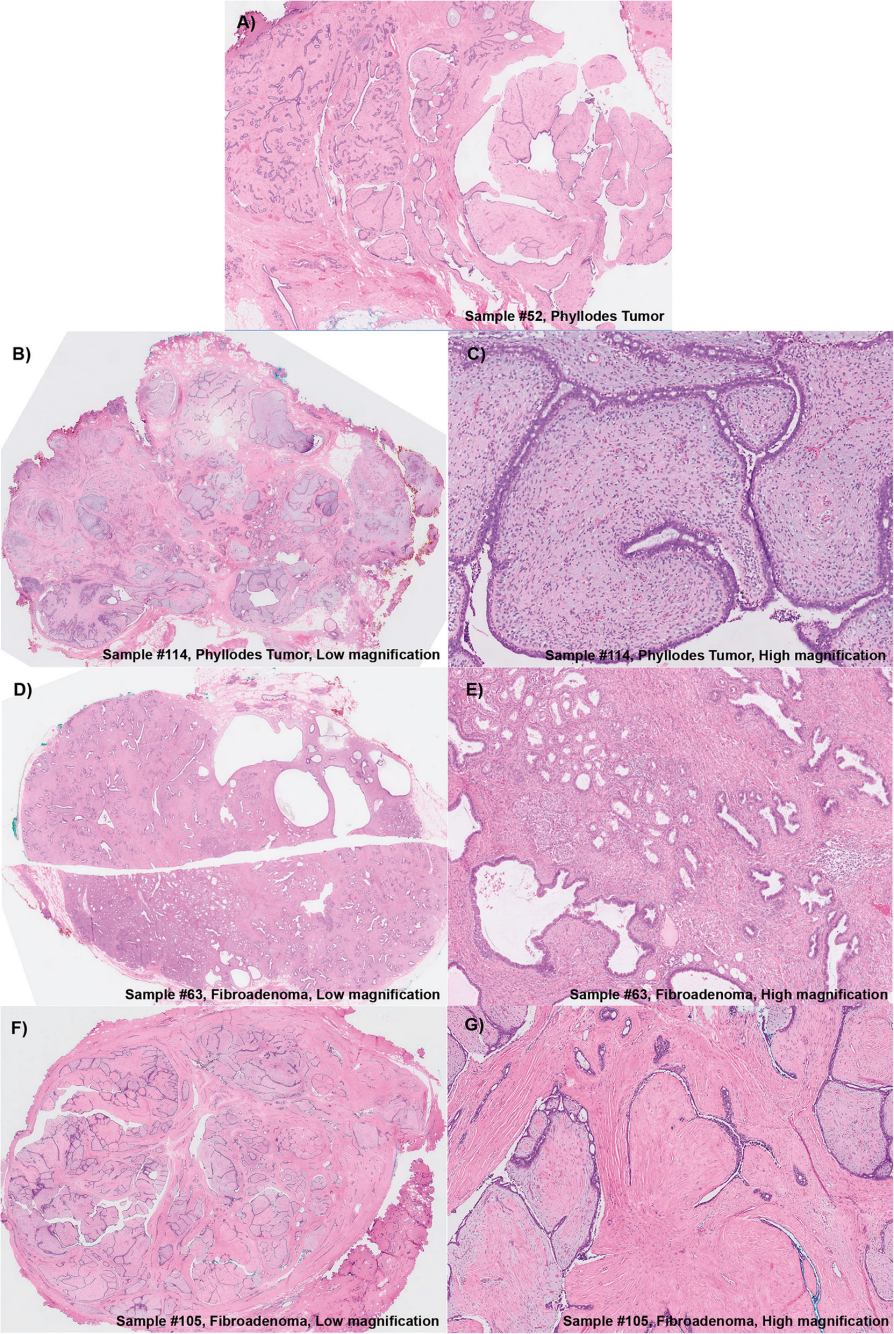
Histological features of the discordant FELs which had their diagnoses changed on second review. a-c Samples 52 and 114 were FA samples upgraded to Benign PTs on review. (D-G) Samples 63 and 105 were PT samples which were downgraded to FAs. **a** Sample 52. Benign fibroepithelial tumor showed well-formed stromal fronds, favoring a benign phyllodes tumor. This was originally diagnosed as a fibroadenoma. **b** Sample 114. Low magnification view of the excised fibroepithelial tumor, with areas showing stromal fronds. **c** Sample 114. On higher magnification, the stromal fronds showed slightly increased cellularity, with peri-epithelial accentuation, in keeping with a benign phyllodes tumor. **d** Sample 63. Low magnification view showed a benign fibroepithelial tumor with areas of adenosis and cysts, accompanied by increased stromal cellularity which appeared diffuse. **e** Sample 63. Higher magnification affirmed the increased stromal cellularity which had a fascicular pattern, with the epithelial component observed as dilated ducts and crowded benign bilayered glands. Upon histological review, this case was regarded as more in keeping with a complex cellular fibroadenoma with cysts. **f** Sample 105. Low magnification view of a benign fibroepithelial tumor, with some clefts (left field) suggesting the presence of fronds. However, overall the tumor had hyalinized and slightly myxoid stroma without increased stromal cellularity. **g** Sample 105. Higher magnification showed the intracanalicular pattern of the tumor which was reviewed as favoring a fibroadenoma

The diagnoses of all 211 lesions obtained through the predictive model were subsequently compared against the pathological reports, and 27 discordant cases were observed—9 FAs and 18 PTs on the original pathological reports, but were predicted otherwise by the 16-gene panel assay and the predictive scoring system (Fig. 4). Six of these nine FAs that were identified as PTs by the model, had possessed a *TERT* promoter mutation and other mutations in other genes such as *FLNA*, *KMT2D*, *MED12*, *RARA*, *RB1* and *SETD2* (Fig. 4). Subsequent pathological review of these nine FAs yielded an upgrade of two FAs (Sample #52 and #114) to benign PTs (Figs. 5a, b, and c). While three samples (#8, #73 and #178) were also noted to have increased cellularity, their original pathological diagnoses of FAs were upheld. In addition, a clinical follow up of these nine patients showed clinical and radiological stability for at least two years, thereby providing support to the original histological diagnoses of FAs. The only exception was the patient (with sample #114) who had a recurrence of a benign PT, and this is concordant with the revised pathological diagnosis. On the contrary, the 18 lesions that were diagnosed as PTs in the pathological reports, were reported as FAs by our predictive model due to the lack of *TP53* mutations and/or a promoter and/or a nonsense mutation. However, they had mutations in other genes such as *EGFR*, *FLNA*, *KMT2D*, *MED12*, *RARA*, *RB1*, *SETD2* and *PIK3CA* (Fig. 4). Subsequent pathological review of these PTs yielded a downgrade of three PTs (Sample #63, #105 and #168) to FAs (Figs. 5d, e, f and g). Together, the 16-gene assay and the predictive model had an accuracy of 89.6%, a specificity of 95.8%, a sensitivity of 65.1%, a positive predictive value (PPV) of 80.0% and a negative predictive value (NPV) of 91.5%.

## Discussion

In this study, we successfully extracted DNA of good quality and quantity from archival FFPE specimens of up to ten years of age, despite the known damage of long term formalin fixation to DNA. Formaldehyde, the main constituent of formalin, is known to generate crosslinks between intracellular macromolecules, forming protein-protein, protein-DNA structures, DNA-formaldehyde adducts and inter-strand DNA crosslinks. DNA fragmentation has also been observed to worsen with the lower pH of formalin used during tissue fixation and the longer storage period of FFPE specimens. Formaldehyde also readily oxidizes with the atmospheric oxygen to form formic acid, encouraging the formation of abasic sites in DNA. Formalin fixation causes cytosine deamination which results in the formation of C > T and G > A sequencing artefacts, thereby leading to the increase in single-nucleotide variant (SNV) rate [28–31]. Hence, to minimize sequencing artefacts that could complicate analysis, strict criteria in the preparation and quality control of FFPE extracted DNA had to be observed, using only biopsies with good quality DNA of sufficient quantity for downstream NGS. Good quality DNA was extracted from 81.3% of the core biopsies and 94.1% of the surgical excisions (Fig. 2a). Although it appears that surgical excisions were a better source of good quality DNA, most of the surgical excisions were “newer” FFPE tissues and hence could have been subjected to lesser degradation by formalin (Fig. 2c). Furthermore, the surgical excisions were larger than core biopsies, offering greater amounts of tissue for gDNA extraction [30]. With minimal DNA degradation expected from ‘fresher’ specimens, a greater yield of DNA with good quality and quantity from an even smaller volume of tissue should be expected for successful downstream application of NGS.

In this study, *MED12* was identified as the most frequently mutated gene amongst FELs (Table 2) [15, 16, 32–36], with observed frequencies similar to those reported in our previous studies [13, 14]. The presence of a common mutation in the *MED12* gene in both FAs and PTs suggests that they share a similar origin, with the comparable frequencies emphasizing their close molecular relationship [14, 32], and that *MED12* aberrations occur early in the pathogenesis of these tumors.

Mutations in the *TERT* promoter were the second commonest mutation observed in PTs but were rare in FAs (Table 2) [17]. Our study showed a seemingly increasing trend from benign to borderline PTs, suggesting that *TERT* promoter mutations may drive the progression of PTs [18]. However, this increasing trend did not continue beyond the borderline PTs to the malignant PTs. This may be due to a bias of the small number of malignant PTs studied, and the possibility that a subset of malignant PTs derived from “*TERT* mutation negative precursors” were included in this analysis [17]. Borderline and malignant PTs of these “*TERT* mutation negative precursors” lacked mutations in the *MED12* gene but mutations were present in *SETD2*, *PIK3CA*, *BCOR*, *NF1*, *EGFR*, *RB1* and *TP53* instead (Fig. 3). It is possible that these PTs arose de novo, via a *MED12* independent pathway, and that these additional mutations were sufficient for acquisition of higher grade PT phenotypes, even in the absence of mutations in both *MED12* and *TERT* promoter [15, 17, 37].

In addition to the *TERT* promoter, mutations in *RARA*, *FLNA*, *RB1* and *TP53* were more frequently observed in PTs than FAs (Table 2), supporting the notion that these genes could drive further progression of FAs to PTs, and may be useful in the discrimination of the two entities [17]. Furthermore, from this select 16-gene assay, a significantly greater number of mutations was observed in PTs compared to FAs (Table 3), consistent with the theory of a stepwise progression of FELs [37]. We were unable to demonstrate a significant difference in the number of mutations observed amongst the different PT grades due to the small number of PTs studied. However, from a clinical perspective, the utility in differentiating FAs from PTs (regardless of the grade of PTs) from a core biopsy is a very useful first step in facilitating the surgeon’s approach to managing a patient with a breast FEL.

The types of mutations found in PTs differed from the FAs. Apart from the missense mutations that were commonly found across all FELs, PTs were more frequently associated with ‘damaging’ mutation types, such as the promoter, frameshift, nonsense and splice site mutations (Table 4). We surmise that these mutation types may have more deleterious effects on protein functions and downstream pathways. Though the relationship between the types of mutations and the malignant potential of a tumor is unclear [33], it is possible that both the genes involved and the types of genetic mutations observed may work synergistically in FEL pathogenesis.

Despite the small numbers of paired biopsies analyzed, we have gained a few insights. Mutations in the paired biopsies were fairly concordant (Fig. 3), validating the 16-gene panel on core biopsy specimens with their corresponding surgical excisions. Though the amount of gDNA available from the core biopsies was limited, we were able to identify additional mutations in two of three paired biopsies. In one pair, the additional mutation (*NF1*) observed in the core biopsy of a malignant PT was present in its corresponding surgical excision, but the variant allelic frequency of this mutation fell beneath the specified threshold of 5% and was excluded. This suggests that the assay was sufficiently sensitive in identifying mutations present in core biopsies that might otherwise be missed in a larger tumor specimen. On the contrary, the other paired biopsies of a benign PT had an additional mutation (*KMT2D*) in the surgical excision specimen and not in the core biopsy (Fig. 3). This additional mutation present in the surgical excision could be due to the core biopsies reflecting only a small part of the tumor and may under-represent the clonal composition of a heterogeneous tumor [38]. The possibility of under-sampling from a core biopsy is a known caveat, and it emphasizes the importance for the clinician in submitting core biopsies that are a good representation of the entire tumor. Due to the retrospective nature of this study and the clinical management of FELs (wide excisions for PTs and surveillance/enucleation for FAs), the procurement of surgical excisions and matched normal tissues is limited for FAs, precluding a more robust genomic comparison between core and excision biopsy material.

Statistical analyses have narrowed down a list of potential predictors that are useful in constructing a predictive scoring system capable of differentiating PTs from FAs. A multivariate regression model was performed on these top ten predictors, identifying three that could work together to classify the FELs. However, with only three predictors in the final model, the scores of these FELs were rather homogenous. It is likely that this relatively small sample size of PTs, a reflection of its low incidence in the clinical setting, may have resulted in the exclusion of other predictors in the final model, due to the lack of significance in multivariate analysis. Nevertheless, the logistic regression results had shown that this predictive model is still competent in predicting PTs (*p* <  0.001).

Our 16-gene assay and predictive model are comparable to the recently available, commercially developed, molecular FibroPhyllo™ Tissue Test [12, 39], with an accuracy of 89.6% (vs 92.6% with FibroPhyllo™ Tissue Test), a specificity of 95.8% (vs 94.7%), PPV of 80.0% (vs 77.3%) and NPV of 91.5% (vs 96.2%) [12]. Despite the lower sensitivity of our model 65.1% (vs 82.9%), this 16-gene panel has acknowledged the biological relevance to FEL pathogenesis, identified and assisted in the reclassification of five FELs. This demonstrates its potential as an adjunctive diagnostic tool in clinical practice, not just in post-operative specimens [16, 23, 24], but particularly pre-operatively, in those core biopsies with indeterminate diagnoses. In addition, this 16 gene panel assay gives detailed information on the genetic mutations present within the tumour, and hence has potential to help differentiate between the different grades of Phyllodes tumours. Validation of this assay on a larger population of PTs is required, and the possible inclusion of other predictors in the final model may help increase the sensitivity and accuracy.

## Conclusion

To our best knowledge, this is the first study to have successfully demonstrated the application of a novel 16-gene assay to reveal mutations across the FEL spectrum on archival FFPE core biopsy specimens. While *MED12* remains the most commonly mutated gene amongst FELs, the *TERT* promoter is the second most frequently mutated gene in PTs. Higher frequencies of mutations observed in the *TERT* promoter, *RARA*, *FLNA*, *RB1* and *TP53* in PTs emphasized the potential of the 16-gene panel in discriminating FEL subtypes. We have also created a preliminary predictive scoring system that classified FELs on core biopsy with good discrimination and calibration. The predictive scoring system categorizes a FEL at low risk (score <  1) or high risk (score ≥ 1) of being a PT, and is significant in predicting PTs (*p* <  0.001). Further work is needed on a larger number of FELs, particularly PTs, to validate this predictive model. We propose that this gene assay and predictive model have the potential to be used in the clinical setting as an adjunctive tool in the diagnosis of FELs on core biopsies, particularly for indeterminate FELs.

## Data Availability

The datasets generated and/or analysed during this current study are available from the corresponding author on reasonable request.

## Abbreviations

BDR: Borderline
BEN: Benign
FA: Fibroadenoma
FEL: Fibroepithelial lesion
FFPE: Formalin-Fixed and Paraffin-Embedded (FFPE)
gDNA: Genomic DNA
MAL: Malignant
NGS: Next Generation Sequencing
PT: Phyllodes Tumor
